# An assessment of functioning and non-functioning distractors in multiple-choice questions: a descriptive analysis

**DOI:** 10.1186/1472-6920-9-40

**Published:** 2009-07-07

**Authors:** Marie Tarrant, James Ware, Ahmed M Mohammed

**Affiliations:** 1 Department of Nursing Studies, Li Ka Shing Faculty of Medicine, 21 Sassoon Road, Hong Kong, PR China; 2 Centre of Medical Education, Faculty of Medicine, Health Sciences Centre, Kuwait University, PO Box 24923, Safat, 13110, Kuwait

## Abstract

**Background:**

Four- or five-option multiple choice questions (MCQs) are the standard in health-science disciplines, both on certification-level examinations and on in-house developed tests. Previous research has shown, however, that few MCQs have three or four functioning distractors. The purpose of this study was to investigate non-functioning distractors in teacher-developed tests in one nursing program in an English-language university in Hong Kong.

**Methods:**

Using item-analysis data, we assessed the proportion of non-functioning distractors on a sample of seven test papers administered to undergraduate nursing students. A total of 514 items were reviewed, including 2056 options (1542 distractors and 514 correct responses). Non-functioning options were defined as ones that were chosen by fewer than 5% of examinees and those with a positive option discrimination statistic.

**Results:**

The proportion of items containing 0, 1, 2, and 3 functioning distractors was 12.3%, 34.8%, 39.1%, and 13.8% respectively. Overall, items contained an average of 1.54 (SD = 0.88) functioning distractors. Only 52.2% (n = 805) of all distractors were functioning effectively and 10.2% (n = 158) had a choice frequency of 0. Items with more functioning distractors were more difficult and more discriminating.

**Conclusion:**

The low frequency of items with three functioning distractors in the four-option items in this study suggests that teachers have difficulty developing plausible distractors for most MCQs. Test items should consist of as many options as is feasible given the item content and the number of plausible distractors; in most cases this would be three. Item analysis results can be used to identify and remove non-functioning distractors from MCQs that have been used in previous tests.

## Background

Single best-answer multiple-choice questions (MCQs) consist of a question (*the stem*), two or more choices from which examinees must choose the correct option (*the distractors*) and one correct or best response (*the key*) [1]. The MCQ format allows teachers to efficiently assess large numbers of candidates and to test a wide range of content [2,3]. If properly constructed, MCQs are able to test higher levels of cognitive reasoning and can accurately discriminate between high- and low-achieving students [2,4]. It is widely accepted, however, that well-constructed MCQ items are time consuming and difficult to write [5]. Furthermore, there is more to writing good MCQs than writing good questions. One aspect where many MCQs fail is in having effective distractors. Teachers often spend a great deal of time constructing the stem and much less time on developing plausible options to the correct answer. High quality MCQs, however, also need the options to be well written [6]. In a classroom setting where test items are designed to measure educational outcomes, distractors must perform acceptably and each distractor should be based on a common misconception about the correct answer [7]. Non-functioning distractors are options that are selected infrequently (<5%) by examinees or otherwise do not perform as expected. As such, these options should be removed from the item [6] or be replaced with a more plausible option. In their review of functioning distractors in 477 items on four MCQ assessments, Haladyna and Downing [7] found that over 38% of distractors on the tests were eliminated because <5% of students selected them. Overall, the percentage of items with three functioning distractors ranged from only 1.1 to 8.4% of all items.

Because a large proportion of distractors in MCQs are non-functioning, determining the optimal number of options in MCQs has been widely investigated. Although four- and five-option items continue to be the standard on teacher-generated tests used to assess students in nursing, medicine, and other health-science disciplines, over the years numerous theoretical [8-10] and empirical research studies [1,7,11-20] have advocated the adoption of three-option MCQs. Research comparing three-option MCQ tests with five-option tests has found that the psychometric properties of the tests are similar and there is no reduction in the reliability or validity of a test when the number of options is reduced [12,15,16,18,19,21]. The benefits of writing fewer options are less test development time, shorter tests, or more items per test to increase sampling of content [7]. Students overwhelmingly prefer items with fewer options [16]. Additionally, for students who speak English as a second language, the benefits of fewer options and shorter reading time will likely be greater.

Despite an existing body of research evaluating the optimal number of distractors in multiple-choice items, substantially less research has focused on examining non-functioning distractors in MCQs in general [7] and no recent studies have specifically examined the frequency of non-functioning distractors in teacher-generated items. Owen and Froman [16] have suggested that items developed by teachers in standard classroom settings be studied further. Examining distractor performance in teacher-generated tests is of interest because the majority of tests students take are teacher-generated and teachers spend a large amount of time developing test items. If this time can be reduced, this is of great practical significance to teaching faculty. Additionally, there is a need for more research on the distractor performance in multiple-choice tests from different perspectives, including observational and item analytic perspectives [8].

## Study Aim

The purpose of this study was to investigate non-functioning distractors in teacher-developed tests to provide additional guidance to teachers in health-science disciplines regarding the optimal number of distractors to include in MCQs. Specifically, we sought to:

1. assess the frequency of functioning distractors in multiple-choice tests;

2. assess the relationship between the number of functioning distractors per item and item psychometric characteristics; and

3. assess the impact of reducing the number of options from four to three on test scores, pass rates, and test reliability.

## Methods

As part of a larger research project [22,23] examining the quality of MCQs in one undergraduate nursing programme in an English-language university in Hong Kong, we retrieved all tests containing MCQs that were administered in clinical and non-clinical nursing courses over a five year period from 2001 to 2005 (n = 121). Test content included basic undergraduate clinical and non-clinical nursing courses taught by 11 different nursing faculty members who also developed the tests. All tests were developed using a test blueprint that maps each test item to the corresponding course objective and are reviewed by a panel of teachers prior to administration. For this analysis, we selected discipline-specific summative tests with a minimum of 50 items, test reliability >.70, and item analysis data available (n = 7). From these seven tests, 514 four-option MCQ items were available for analysis. All tests were criterion-referenced and pass scores were set at 50%.

Previous studies have used various methods for evaluating distractor quality, including response frequency (non-functioning is usually defined as <5%) [1,7,14,17,18,21], poor distractor discrimination [13,16,19], expert judgment [1,15,24], and examination of option characteristic curves (trace lines) [7]. Trace lines graphically display the response patterns of the item options but typically require a large sample of examinees (200+) [25]. Evaluating distractor quality using expert judgment is more commonly used in building items and is not required when item analysis data is available, as it was in this study. Therefore, for this study we used the first two criteria to evaluate distractor performance. First, a non-functioning option was defined as one that was chosen by fewer than 5% of examinees. Second, we assessed the discriminating power of the options. Discriminating power is an index that measures the difference in the proportion of responses between the upper and lower 27% of examinees [26]. Items are considered discriminating if the index for the correct response is positive and the same statistic for the distractors is negative [25]. From the item-writers perspective, good distractors appeal to a higher proportion of low-achieving examinees when compared with high-achieving examinees, thereby resulting in a negative statistic [7]. The advantage of the discrimination index is that it is simple to compute and explain [26]. Therefore, a non-functioning distractor was defined as an option with either a response frequency of <5% or a positive discriminating power.

### Data Analysis

Frequency distributions were constructed for all 514 items, which included 2056 options (1542 distractors and 514 correct responses). Item difficulty is the proportion of examinees answering the question correctly, with lower values reflecting more difficult questions [25]. All distractors with a choice frequency of <5% were identified. We further computed the discriminating power of all distractors and identified distractors with positive discriminating power (non-functioning distractors). We constructed frequency distributions for the number and proportion of: (1) distractors with low choice frequency (<5%), positive discrimination, and 0% choice frequency; (2) functioning distractors per test; and (3) items with 0, 1, 2, and 3 functioning distractors. We computed the mean number of functioning distractors per item and then further assessed the relationship between the item difficulty and the point-biserial correlation coefficient and the number of functioning distractors per item using analysis of variance (ANOVA) statistics. The point-biserial correlation coefficient measures the association between the test item and the total test score [25]. Finally, product moments correlation coefficients (Pearson's *r*) were computed between the item difficulty and the point-biserial correlation coefficient statistics and the number of functioning distractors per item.

To assess the impact of reducing the number of options from four to three, we first removed all distracters with a choice frequency of zero. Then, for each item with four remaining options, the option with the lowest choice frequency was randomly redistributed to the remaining three options. The random redistribution was based on the assumption that those examinees who choose the least popular distractor are likely guessing and therefore random redistribution would legitimately reflect the process of choice selection for these examinees if three options were presented instead of four. We then assessed the impact of this redistribution on test scores and test reliability.

Item-analysis was conducted using Ideal 4.1, an item-analysis program (IDEAL-HK, Hong Kong, China) [27] and all other data analysis was conducted using Stata version 9.2 (Stata Corporation Inc., College Station, TX, USA) [28]. This study was exempted from ethical review by the Institutional Review Board of the University of Hong Kong because it did not involve human subjects' data.

## Results

Table 1 shows the characteristics of the assessed tests. The number of items on the tests ranged from 50 to 100 while the number of examinees ranged from 73 to 146. Mean test scores ranged from 55.5% to 72.0% and the reliability of the tests, as measured by the Kuder Richardson (KR) 20, ranged from .70 to .87 with tests having a higher number of items generally being more reliable.

**Table 1:** Characteristics of the Tests

	Test A	Test B	Test C	Test D	Test E	Test F	Test G	Total
No. of items	96	72	86	50	50	60	100	514
No. of examinees	146	74	74	73	73	73	75	588
Mean test score % (SD)	67.7 (9.87)	55.5 (8.52)	69.2 (10.44)	72.0 (10.82)	62.6 (11.28)	67.8 (10.02)	65.6 (11.29)	--
Range of test scores (%)	38–89	33–71	38–90	46–94	34–88	35–88	34–89	--
KR20 Reliability	.81	.71	.82	.71	.72	.70	.87	--

SD = standard deviation; KR-20 = Kuder-Richardson 20

Overall, 514 items and 1542 distractors were assessed (Table 2). 541 distractors (35.1%) had a choice frequency of <5% and 472 (30.6%) distractors had positive discrimination statistics; 17.9% (n = 276) of infrequently selected distractors were also non-discriminating. A substantial proportion of distractors were so implausible (10.2%) they were not chosen by anyone. Just over one-half (52.2%) of all distractors were classified as functioning. The proportion of items with three functioning distractors ranged from 5.0% on Test F to 24.0% on Test E (Table 2). Overall only 13.8% of items had three functioning distractors and 12.3% had no functioning distractors. The mean number of functioning distractors per item ranged from 1.35 (SD = .82) on Test B to 1.74 (SD = .86) on Test A and Test E (SD = .96). The overall mean number of functioning distractors per item was 1.54 (SD = .88). Items with only 0 or 1 functioning distractors were significantly less difficult than items with 2 or 3 functioning distractors (Table 3). On four of the tests, items with two functioning distractors were more difficult than items with three functioning distractors. Items with more functioning distractors were uniformly more discriminating than those with fewer functioning distractors (Table 4).

**Table 2:** Distractor Performance

	Test A	Test B	Test C	Test D
No. of items	96	72	86	50
No. of distractors assessed	288	216	258	150
Distractors with:				
Frequency <5% n(%)	88 (30.6)	78 (36.1)	109 (42.2)	60 (40.0)
Discrimination ≥ 0	68 (23.6)	75 (34.7)	88 (34.1)	48 (32.0)
Both	40 (13.9)	39 (18.1)	60 (23.3)	34 (22.7)
Frequency = 0% n(%)	12 (4.2)	24 (11.1)	42 (16.3)	21 (14.0)
Functioning distractors per test n(%)	172 (59.7)	102 (47.2)	121 (46.9)	76 (50.7)
Functioning distractors per item n(%)				
None	8 (8.3)	11 (15.3)	13 (15.1)	7 (14.0)
One	27 (28.1)	30 (41.7)	35 (40.7)	16 (32.0)
Two	43 (44.8)	26 (36.1)	28 (32.6)	22 (44.0)
Three	18 (18.8)	5 (6.9)	10 (11.6)	5 (10.0)
Functioning distractors per item M(SD)	1.74 (.86)	1.35 (.82)	1.41 (.89)	1.50 (.86)
				
	
	Test E	Test F	Test G	Total
	
No. of items	50	60	100	514
No. of distractors assessed	150	180	300	1542
Distractors with:				
Frequency <5% n(%)	43 (28.7)	62 (34.4)	101(33.7)	541 (35.1)
Discrimination ≥ 0	46 (30.7)	69 (38.3)	78 (26.0)	472 (30.6)
Both	26 (17.3)	33 (18.3)	44 (14.7)	276 (17.9)
Frequency = 0% n(%)	15 (10.0)	21 (11.7)	23 (7.7)	158 (10.2)
Functioning distractors per test n(%)	87 (58.0)	82 (45.6)	165 (55.0)	805 (52.2)
Functioning distractors per item n(%)				
None	6 (12.0)	8 (13.3)	10 (10.0)	63 (12.3)
One	13 (26.0)	25 (41.7)	33 (33.0)	179 (34.8)
Two	19 (38.0)	24 (40.0)	39 (39.0)	201 (39.1)
Three	12 (24.0)	3 (5.0)	18 (18.0)	71 (13.8)
Functioning distractors per item M(SD)	1.74 (.96)	1.37 (.78)	1.65 (.89)	1.54 (.88)

**Table 3:** Relationship between Number of Functioning Distractors and Item Difficulty

	None	One	Two	Three	*r*
Test A	.94	.77	.60	.60***	-.51***
Test B	.71	.54	.48	.67	.13
Test C	.88	.71	.60	.62***	-.44***
Test D	.93	.72	.66	.67**	-.46***
Test E	.78	.71	.59	.51*	-.43**
Test F	.77	.72	.63	.55	-.33**
Test G	.89	.70	.57	.62***	-.34***

* *p *< .05

** *p *< .01

*** *p *< .001

**Table 4:** Relationship between Number of Functioning Distractors and Point-Biserial Correlation Coefficient

	None	One	Two	Three	*r*
Test A	.09	.19	.25	.28***	0.44**
Test B	.06	.18	.24	.36*	0.35**
Test C	.10	.22	.31	.36***	0.53***
Test D	.17	.21	.28	.40*	0.45**
Test E	.09	.12	.33	.38***	0.64***
Test F	.14	.23	.26	.38	0.32*
Test G	.24	.22	.29	.36*	0.28**

* *p *< .05

** *p *< .01

*** *p *< .001

Results of the redistribution of poor functioning distractors are presented in Table 5. A total of 384 options were redistributed with 124 (32.3%) reallocated to the keyed option. Approximately 5% of examinees would benefit from the redistribution with 11 (1.9%) examinees being reclassified as pass instead of fail. A comparison of the four-option tests and the three-option tests is presented in Table 6. Mean test scores increased from +0.6% to +1.8%. There were minimal changes in the range of test scores and test reliability.

**Table 5:** Results of Reallocation of Poor Functioning Distracters

	Test A	Test B	Test C	Test D	Test E	Test F	Test G	Total
Redistributed options n	88	49	57	30	37	44	79	384
Redistribution to keyed option n	30	7	20	13	12	21	21	124
Examinees benefiting %	4.61	7.34	4.26	4.43	5.02	4.89	5.46	--
Examinees re-classified as pass n	1	3	2	1	2	1	1	11

**Table 6:** Comparison of Four-Option and Three-Option Tests Generated by Random Redistribution of Fourth Option

	Mean test score % (SD)		Range of test scores (%)		KR20 Reliability	
	
	4-options	3-options	4-options	3-options	4-options	3-options
Test A	67.4 (10.04)	69.2 (9.46)	38–90	38–90	.81	.80
Test B	55.4 (8.84)	56.1 (8.61)	33–71	35–71	.71	.70
Test C	69.2 (10.44)	70.1 (9.86)	38–90	41–90	.82	.79
Test D	71.9 (10.82)	72.5 (10.70)	46–94	46–94	.71	.69
Test E	64.2 (8.35)	64.9 (8.21)	46–84	46–84	.73	.70
Test F	67.9 (10.02)	69.1 (9.79)	35–88	35–90	.70	.69
Test G	65.6 (11.29)	66.6 (10.80)	34–89	36–89	.87	.85
Total (mean)	(65.9)	(66.9)	--	--	(0.76)	(0.75)

## Discussion

Results from this study show only 13.8% of all items had three functioning distractors and just over 70% had only one or two functioning distractors. The low proportion of items with three functioning distractors was not altogether surprising given that all tests were generated by in-house teaching faculty, most of who have minimal training in item writing – a situation that is likely similar to most tertiary education settings. Furthermore, other research suggests that even professionally developed test items on standardized exams rarely have more than two functional distractors. Haladyna and Downing [7] found that approximately two-thirds of all four-option items they reviewed had only one or two functioning distractors and none of the five-option items had four functioning distractors. Because it is often difficult for teachers to develop three or more equally plausible distractors, additional distractors are often added as "fillers." An item with two plausible distractors, however, is preferable to an item with three or four implausible distractors [4,13] as students rarely select these options anyway. More is not necessarily better when producing distractors – the key is the quality of the distractors, not the number [6]. The low frequency of items with more than two functioning distractors and the finding that only about one-half of all distractors were functioning suggests that three-option items are the most practical choice for in-house tests. Haladyna and Downing [7] concluded that because so few items had more than two functioning distractors, "three options may be a natural limit for multiple-choice item writers in most circumstances" (p. 1008). A meta-analysis of 80 years of research on the number of options in MCQs also concluded that three options is optimal for MCQs in most settings [29].

Conversely, there is no psychometric reason that all items must have the same number of options as some questions would naturally have more or less plausible distractors than others [30]. So while in most circumstances, three options would be sufficient, item writers should write as many good distractors as is feasible given the content area being assessed [6,18]. Additionally, when reviewing item performance on previous tests, test developers and item writers should not eliminate options that perform adequately simply to conform to a pre-set number of options [16]. Many teacher-developed tests however, particularly summative tests, must conform to institutional guidelines as to how many options test items have. These guidelines are rarely evidence-based [31] and are more likely to be based on routine practices and/or set procedures. Teachers often do not have the flexibility to set items with varying numbers of options. In such circumstances, given the low proportion of items with four functioning distractors, three-option items would appear to be the most reasonable choice.

Of further concern is the high proportion of items that did not have any functioning distractors (12.3%). These items would inevitably have high item difficulty statistics (>.90) with almost all students getting the items correct. When absolute pass scores are used and set at a fixed percentage (i.e., 50%), as they are in the institution where these tests were administered, such a high proportion of easy items likely results in many borderline candidates passing who should not. Pass standards should be set relative to the difficulty of the test using one of a number of established procedures (i.e, the Angoff method or the Ebel procedure) [32] not simply by using a common but arbitrary figure such as 50%.

Although MCQs with three functioning distractors produced the most discriminating items in this study, this relationship should be viewed with caution as option discrimination and item discrimination are closely related and it is inevitable that items with more discriminating options are more discriminating overall. Items in this study with more functioning distractors were also more difficult than options with fewer functioning distractors. There was, however, little difference in item difficulty between items with two and three functioning distractors. Other research comparing item discrimination and difficulty when the number of options was reduced has found no difference in the shorter items. Owen and Froman [16] randomly administered 100 items to 114 undergraduate students as either five-option items or three-option items and found no significant differences in either item discrimination or difficulty. In comparing five-option items with both three- and four-option items, Trevisan et al. [19] found that three-option items were more discriminating and had fewer items with non-performing distractors than five-option items. A review of numerous studies concluded that reducing items from four options to three options decreases item difficulty (.04), increases item discrimination (.03), and also increases reliability (.02) [29]. Conversely, developing new three-option items without the benefit of knowing how items have already performed may not produce the same improvements in item and test psychometric properties as reducing the number of options in previously tested items [18]. If three-option items are not well constructed and the two available distractors are non-functioning, overall test scores would increase substantially. When developing new items, irrespective of the number of options, items should be developed by content experts in accordance with accepted item writing guidelines and peer reviewed prior to use to ensure that the answer is unambiguously correct and that all distractors are plausible [33].

Despite a growing body of research supporting the use of three-option MCQs, this format continues to be the exception rather than the norm. Large testing bodies [34], item-writing textbooks [16], instructor's manuals and MCQ item banks [35] rely on either four- or five-option MCQs. Hence, most teacher-developed MCQs in health-science disciplines are either four- or five option items. Why teachers have been reluctant to use three-option MCQs is unclear. It may be that longer more complex items appear to be more rigorous [16]. Teachers may also feel that three-option MCQs increase weaker students' chances of guessing the correct option [18]. Furthermore, teaching and assessment practices are often handed down from senior to junior teachers and four- or five-option items are the traditional MCQ format [16]. Finally, it may also be that teachers themselves have little control over the format and type of items used in institutional assessments. These policies may be set by administrators, who for the same reasons identified above, are reluctant to use fewer than four or five options on summative tests.

Three-option MCQs however, offer many benefits to teachers. First, fewer options reduce testing time [6,36]. Conversely, with fewer options, more items can be added to tests to increase the sampling content while keeping testing time constant. Aamodt & McShane [11] estimated that on three-option tests, students can complete an additional 12.4 MCQs in the same time required to complete 100 four-option items. A greater number of items also has the additional benefit of increasing test reliability. Additionally, writing only three-options per item saves time generating items. Generating plausible options is time consuming and if each distractor takes five minutes to generate, writing three-option instead of five-option items will save over 16 hours of time on a 100-item test [18]. Furthermore, our simulated analysis demonstrates that reducing the number of options from four to three does not result in substantially higher scores as a result of guessing. Overall, there was only a 1% increase in mean test scores after removal of the least functioning distractor. The effect of guessing on multiple-choice tests scores is often overestimated and our analysis is consistent with other research which found that on a 100-item test, reducing items from four or five to three-options resulted in a test-score increase of only 1.22 points [11].

Results from this study also highlight the importance of reviewing item performance after test administration and using these results to eliminate non-functioning distractors to improve test items in future administrations of the test. The performance of each test item along with each distractor should be assessed using item analysis procedures. Item analysis procedures involve examining the statistical properties of test items in relation to a response distribution [25]. Distractors that <5% of students select or distractors with discrimination statistics ≥ 0 can easily be identified and modified or removed in future tests. Teachers and test developers can expect that 50% or more of the items they write will fail to perform as expected [37]. Therefore, item analysis provides valuable data for question improvement and should be incorporated into the process of test development and review. It is only through this iterative process of item analysis and improvement that pedagogically and psychometrically sound tests can be developed.

## Strengths and limitations

To our knowledge, this is the first study in a health-science discipline to specifically examine functioning and non-functioning distractors in teacher-generated tests and as such provides a realistic assessment of the limitations of most four- or five-option multiple-choice items. Findings from this study are consistent with the body of research supporting three-option MCQs. Generalizability of the findings from this study, however, may be limited by several factors. First, this study examined functional distractors in tests administered in one nursing programme over a defined period of time. Although we assessed a large number of tests with consistent results, it is possible that the outcomes observed in this study do not reflect teacher-generated MCQs in other academic settings. Additionally, since we did not randomly select our tests, it is also possible that our analysis suffers from some selection bias and that our findings do not accurately reflect the proportion of non-functioning distractors in teacher-generated tests. We also do not have item analysis data on items which may have been used in previous tests. Therefore, we cannot determine what impact, if any, item performance in a previous test may have had on item selection for the tests. As we only used the item psychometric properties from one administration of the test, it is also possible that the options we have identified as non-functioning distractors in these tests are subject to sampling bias and would in fact perform quite differently in other samples. Furthermore, our random redistribution of distractor choice may not simulate choices examinees would actually make when presented with three-options instead of four-options. Examinees may be more likely to engage in educated guessing rather than blind guessing as most students have at least some partial knowledge about the content. Finally, although this study was conducted in an educational setting where English is the medium of instruction (EMI) but not the native language, we were unable to assess the impact of language and reading ability on responses to multiple-choice items. Given the numerous studies that suggest the three-option format is superior to four- or five-option formats in traditional educational settings, we would expect the benefits to be greater in EMI settings. Further research should investigate the impact of reducing the number of options on testing time required for students using English as a second language in academic settings.

## Conclusion

Writing high quality distractors is an important part of the item and test development process. Ideally multiple-choice items should consist of as many options as is feasible given the item content and the number of plausible distractors. Results from this and other studies show that in most circumstances, this will be three options. Because the majority of items developed by teachers will not have more than two functioning distractors, including more distractors may not be a good investment of a teacher's time in item development. Three option-items have many advantages for both item writers and examinees and additional non-functioning distractors are not likely to improve item or test psychometric properties.

## Competing interests

The authors declare that they have no competing interests.

## Authors' contributions

MT and JW contributed equally to the study design. MT obtained funding, performed data analysis and wrote the first draft of the paper. AMM assisted with data analysis. JW and AMM critically reviewed and revised the final draft of the paper. All authors read and approved the final manuscript.

## Pre-publication history

The pre-publication history for this paper can be accessed here:


